# Broad-Spectrum Antimicrobial Activity of a Ruthenium(II) Polypyridyl Complex against Multidrug-Resistant Uropathogens and Biofilms

**DOI:** 10.1021/acsomega.6c03019

**Published:** 2026-06-18

**Authors:** Beth James, Simon D Fairbanks, Mia Horton, Jim A Thomas, Samantha McLean, Adam M Varney

**Affiliations:** 1 Medical Technologies Innovation Facility (MTIF), 6122 Nottingham Trent University, Clifton Lane, Nottingham NG11 8NS, U.K.; 2 Department of Chemistry, 7315 University of Sheffield, Brook Hill, Sheffield S3 7HF, U.K.; 3 School of Science and Technology, 6122 Nottingham Trent University, Clifton Lane, Nottingham NG11 8NS, U.K.

## Abstract

Antimicrobial resistance and biofilm persistence are major barriers to treating device-associated infections, including catheter-associated urinary tract infections. We evaluated KLS-116, a ruthenium­(II) polypyridyl complex, as a next-generation antimicrobial with broad activity against multidrug-resistant uropathogens. KLS-116 showed potent bactericidal effects under physiologically relevant conditions, with minimum inhibitory concentrations ≤ 25 μM (41.8 mg/L) across diverse species. Checkerboard assays demonstrated compatibility with standard antibiotics, yielding indifferent or additive interactions and no antagonism. Importantly, KLS-116 reduced mature biofilms in continuous-flow catheter models and in static models achieved complete clearance at ≥16× MIC after 24 h, outperforming colistin with prolonged exposure. Over 42 days, resistance evolution showed minimal sensitivity shifts, contrasting with rapid ciprofloxacin resistance, indicating a low propensity for resistance. Activity varied with pH, suggesting that the metabolic state influences efficacy. These results position KLS-116 as a promising candidate for CAUTI management and other biofilm-driven infections, guiding future delivery optimization and in vivo validation.

## Graphical abstract

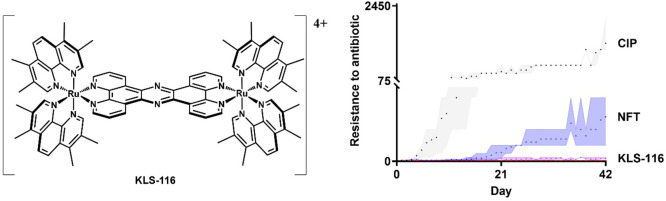

## Introduction

Antimicrobial resistance (AMR) remains one of the most urgent global health threats, which is being driven by the rapid emergence of multidrug-resistant Gram-negative pathogens and the stagnation of the antibiotic development pipeline.^1–3^ The most recent WHO annual pipeline report identifies only 90 new antibacterials or therapeutic combinations including new entities being clinically developed to target the WHO bacterial priority pathogens list or *Clostridioides difficile* and *Helicobacter pylori*.^4,5^ By contrast, there are over 5000 anticancer therapeutics in clinical development highlighting the disparity in innovation for antibacterials.^6^

Infections caused by multidrug-resistant organisms such as *Klebsiella* spp., *Pseudomonas aeruginosa*, and *Acinetobacter baumannii* are associated with high morbidity, mortality, and escalating healthcare costs. These prominent members of the ESKAPE group of pathogens collectively account for a substantial proportion of hospital-acquired infections and exhibit extensive multidrug resistance.^7^ The clinical burden they cause is amplified in device-associated infections, particularly catheter-associated urinary tract infections (CAUTIs), which are some of the most common healthcare-associated infections worldwide. In England alone, CAUTIs cost the NHS an estimated £99 million annually, with an average of £1968 per episode.^8^ Approximately 4.8% of CAUTI patients develop catheter-associated bloodstream infections, which carry a mortality rate of nearly 19.5% and often result in chronic health complications among survivors.^9^ Globally, CAUTIs are a leading contributor to secondary bloodstream infections and are strongly associated with multidrug-resistant organisms, making treatment failure common.^10^ Antimicrobial resistance further exacerbates this burden, with urinary tract infections linked to AMR causing an estimated 260,000 deaths worldwide in 2019.^11^

Indwelling urinary catheters provide a surface for the rapid formation of biofilms, which supply a protective matrix to shelter sessile bacterial communities from host immune responses and antimicrobial agents.^12^ Within biofilms, bacteria exhibit altered physiology, including reduced metabolic activity and stress-adaptive states, thereby conferring extreme tolerance to conventional antibiotics and inducing increased virulence. This frequently leads to treatment failure, often necessitating catheter removal or invasive interventions, thus contributing to prolonged hospitalization, increased risk of systemic infection, and significant economic impact.^13,14^

Given these challenges and the rising prevalence of multidrug-resistant pathogens in device-associated infections, the lack of new antibiotic classes entering clinical practice underscores the urgent need for innovative approaches that maintain efficacy under physiologically relevant conditions. In this context, transition-metal complexes, especially those based on ruthenium­(II), have re-emerged as promising candidates due to their structural versatility, stability, and ability to interact with multiple biological targets.

Early pioneering work by the Dwyer group demonstrated that polypyridyl complexes of ruthenium and other transition metals possess antimicrobial activity against a range of pathogens,^15^ leading to clinical trials of these compounds as topical treatments for skin infections.^16,17^ Subsequent studies by the Collins and Keene groups renewed interest in this class by exploring oligonuclear Ru­(II) and Ir­(III) complexes,^18–20^ which showed strong activity against Gram-positive bacteria but limited efficacy against Gram-negative species.^21–26^ However, few such complexes have progressed beyond early stage evaluation; furthermore, their ability to tackle clinically relevant challenges, such as biofilm eradication and compatibility with existing therapies, remains largely unexplored. Building on this gap, the Thomas group recently derivatized a dinuclear Ru­(II) complex, originally developed as a nontoxic imaging probe for eukaryotic cell nuclei,^27–29^ into a highly active, broad-spectrum antimicrobial, now known as KLS-116 (Figure 1), with potent activity against multidrug-resistant pathogens, including uropathogenic *Escherichia coli*, and demonstrated its capacity to clear ESKAPE infections *in vivo*.^30–32^

**Figure 1:**
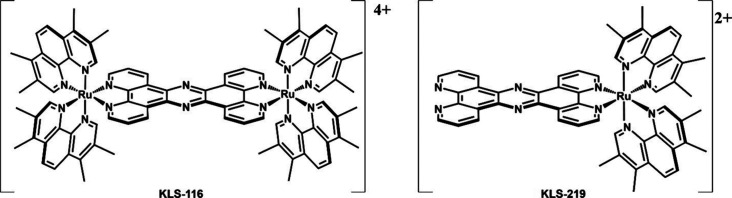
Structures of compounds KLS-116 and KLS-219 studied in this report.

While the antibacterial activity and general mechanism of action of KLS-116 have been reported previously, the present study addresses a distinct and critical question: whether this compound retains efficacy under biologically and clinically relevant conditions that commonly undermine progression of antimicrobial candidates toward *in vivo* application. By systematically evaluating activity in UTI-relevant media, across physiologically relevant pH ranges, and against both early stage and mature catheter-associated biofilms, as well as in the context of resistance evolution, this work aims to derisk translational failure at an early stage and assess the suitability of KLS-116 for further preclinical development.

## Results

### KLS-116 Activity against *E. coli* EC958 Is Modulated by Environmental pH

To progress novel antimicrobials toward clinical application, it is essential to evaluate their performance under conditions that reflect physiological complexity and clinical relevance. This includes assessing activity against diverse pathogens, understanding how environmental factors such as pH influence efficacy, determining compatibility with existing antibiotics, and evaluating biofilm eradication potential. pH was selected as a key variable due to its clinical relevance, as the local pH of urine can vary substantially. It is also known to strongly influence the activity of many clinically used antibiotics. In particular, fluoroquinolones such as ciprofloxacin exhibit reduced antibacterial activity under acidic conditions, which has been attributed to changes in drug ionization state and reduced bacterial uptake at low pH. Similar pH-dependent reductions in efficacy have been reported for aminoglycosides, whose uptake is impaired under acidic conditions due to reduced proton-motive force, as well as for several β-lactam antibiotics, for which stability and bactericidal activity can vary with pH.^33–35^

To determine how environmental pH influences KLS-116 activity, we assessed its efficacy against *E. coli* EC958, a well-characterized multidrug resistant uropathogen previously studied with ruthenium complexes^30,32^ across a physiologically relevant pH range (5–9). Growth in a defined minimal medium containing glucose was optimal at pH 9 and progressively declined under acidic conditions, with minimal growth at pH 5 and no detectable growth observed at pH 3–4.^36^

When cultures were exposed to KLS-116, the compound retained measurable antibacterial activity across the full pH range tested (pH 5–9), with enhanced inhibition observed under mildly alkaline conditions that also support optimal bacterial growth (Figure 2). MIC testing in a pH-adjusted chemically defined medium revealed a consistent increase in susceptibility with rising pH, with MIC values decreasing from 5.9 μM at pH 5 to 1.6 μM at pH 9 (Figure 2A). This trend was mirrored in bactericidal assays, where MBC values similarly declined as pH increased, although bactericidal activity appeared to plateau between pH 5 and 7 (Figure 2B). Collectively, these findings indicate that the antibacterial activity of KLS-116 is relatively robust across environmentally relevant pH conditions and less susceptible to pH-driven variation than many conventional antibiotics. We also noted that MIC and MBC values at a pH just below the major susceptibility shift displayed increased interexperimental variability. This observation suggests that there is a physiological transition point for EC958 at this pH, where growth rates, membrane properties, and ionization state of the compound may fluctuate between experiments, resulting in increased variability prior to the consistent enhancement of activity observed at higher pH. These findings indicate that pH modulates KLS-116 activity; however, across the entire tested pH range, antibacterial activity remained high.

**Figure 2:**
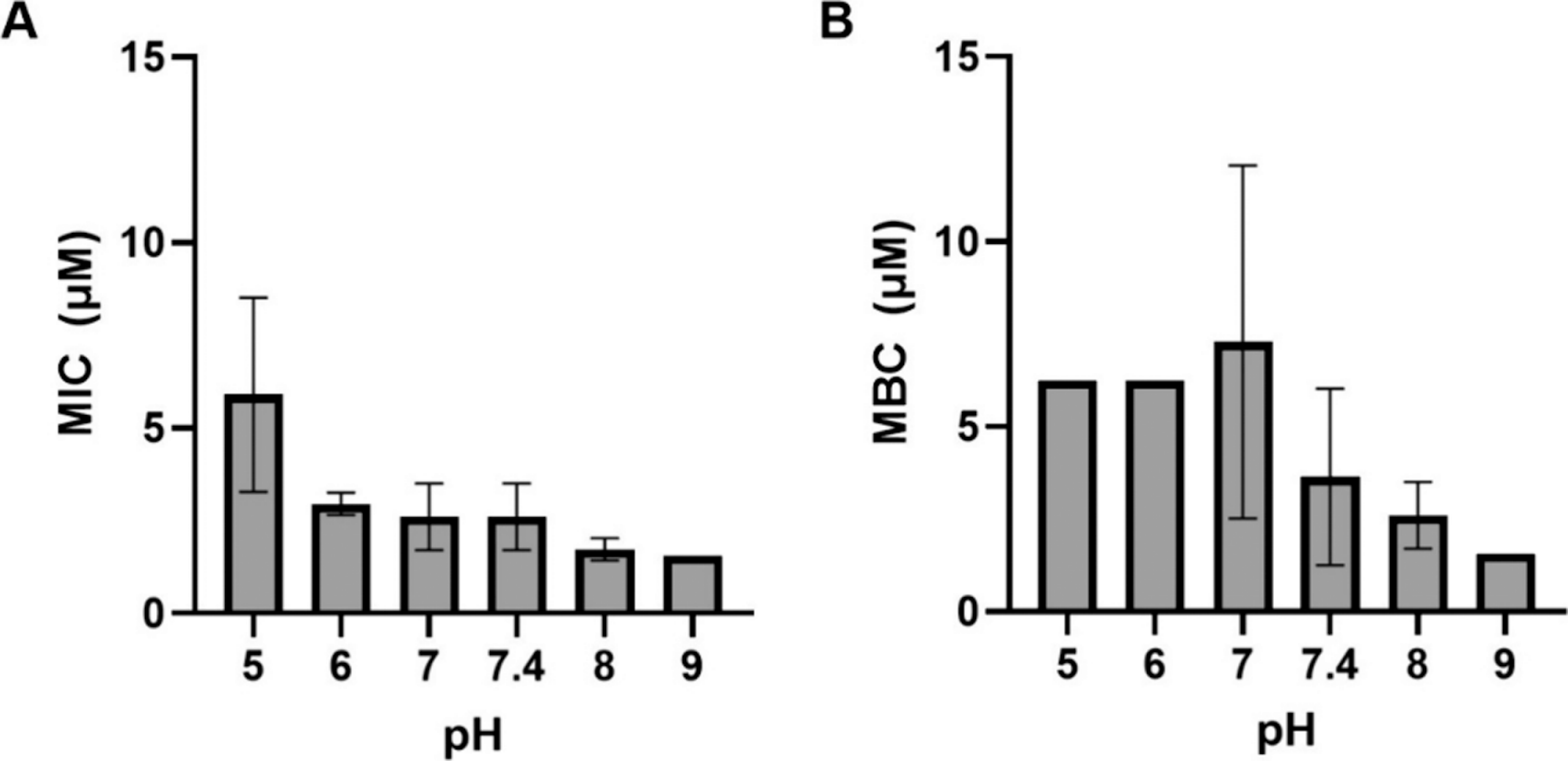
Effect of pH on the antimicrobial activity of KLS-116 against *E. coli* EC958**.** (A) Minimum inhibitory concentrations (MIC) and (B) minimum bactericidal concentrations (MBC) were determined in pH-adjusted chemically defined medium following 18 h of incubation at 37 °C. Bactericidal activity was confirmed by spotting 10 μL from wells without visible growth onto Mueller–Hinton agar and incubating at 37 °C for 24 h. Data represent mean ± SD from three biological replicates.

### Resistance Profile of *E. coli* EC958 to Conventional Antibiotics and Implications for Combination Therapy with KLS-116

Real-world use of new antimicrobials must integrate into current treatment regimens without antagonism; hence, compatibility with existing antibiotics is critical for clinical translation and regulatory approval. To establish a baseline for combination therapy studies, we first confirmed the antibiotic susceptibility of *E. coli* EC958 in accordance with EUCAST guidelines.^37^ The strain exhibited high-level resistance to the β-lactams ampicillin and cephalexin, as well as the fluoroquinolone ciprofloxacin, while remaining sensitive to Meropenem. Minimum inhibitory and bactericidal concentrations for these antibiotics were determined in both Mueller–Hinton broth (MHB) and a glucose defined minimal medium (gDMM) to ensure compatibility with KLS-116 testing (Table 1). As expected, resistance was more pronounced in MHB; however, all three resistant antibiotics showed increased activity in gDMM, with ciprofloxacin demonstrating the greatest shift (16-fold reduction in MIC), followed by cephalexin (4-fold) and ampicillin (2-fold). Although the strain remained sensitive to Meropenem in both media, an 11-fold MBC increase in the defined medium compared with MHB was observed.

**Table 1:** Susceptibility of *E. coli* EC958 to Conventional Antibiotics and Interaction Profiles with KLS-116 in Combination Therapy^a^

	media						
	gDMM		MHB				
antibiotic	MIC	MBC	MIC	MBC	S/R	KLS-116 FIC Index	s/ad/in/an
cephalexin	128 (0.00)	383 (0.34)	512 (0.00)	2048 (0.00)	R	1.03 (0.00)	indifference
ampicillin	2048 (0.00)	4096 (0.00)	4096 (0.00)	>4096 (0.00)	R	1.02 (0.01)	indifference
ciprofloxacin	256 (0.00)	4096 (0.00)	4096 (0.00)	>4096 (0.00)	R	0.81 (0.18)	additive
Meropenem	0.046 (0.01)	0.688 (0.00)	0.031 (0.01)	0.059 (0.01)	S	0.62 (0.10)	additive

a s – synergistic, ad – additive, in – indifferent, an – antagonistic,. EUCAST breakpoints used to determine clinical susceptibility or resistance (S – sensitive, R – resistant).^38^ Data is a mean of three biological repeats (MIC/MBC) and six biological repeats (checkerboard) ± SD in brackets.

Using these data as a reference, we performed checkerboard assays to evaluate potential interactions between KLS-116 and the four antibiotics. All assays were conducted in the chemically defined medium to maintain consistency with prior MIC and MBC determinations. All KLS-116–antibiotic combinations exhibited either indifference or additive effects, with fractional inhibitory concentration (FIC) indices ranging from 0.62 to 1.03 (Table 1). Importantly, no antagonistic interactions were observed. The combination of KLS-116 and Meropenem yielded the lowest FIC index (0.62), approaching the threshold for synergy (≤0.5).

These findings indicate that KLS-116 does not compromise the activity of commonly used antibiotics and may be suitable for inclusion in combination therapies, particularly in clinical scenarios involving multidrug-resistant infections or antimicrobial coatings on medical devices.

### Resistance Evolution Assays Demonstrate Low Propensity for Resistance Development to KLS-116

To address a critical requirement for advancing new antimicrobials toward clinical use, that resistance does not emerge rapidly, we performed an evolution of resistance assay in *E. coli* EC958 as it provides a relevant model for assessing long-term resistance trajectories. Cultures were exposed to a six-well, 2-fold dilution gradient (0.25× to 8× MIC of each compound), with MICs reassessed every 24 h and drug concentrations adjusted accordingly to maintain selective pressure. This process was sustained over 42 consecutive days across five independent biological replicates under each condition. For comparison, parallel assays were conducted using two antibiotics with well-characterized resistance trajectories, ciprofloxacin and nitrofurantoin, as well as KLS-219 (Figure 1), the mononuclear analogue of KLS-116.

Over the 42-day period, no significant increase in MIC was observed for either of the KLS complexes (Figure 3), in contrast to the marked resistance development seen with the comparator antibiotics. Specifically, ciprofloxacin resistance increased by approximately 1024-fold, while nitrofurantoin resistance rose by ∼32-fold. Notably, a transient 2-fold increase in MIC was observed in some KLS-116-evolved lineages; however, this reverted to baseline following strain storage and reculturing, suggesting instability or lack of fixation of resistance-conferring mutations.

**Figure 3:**
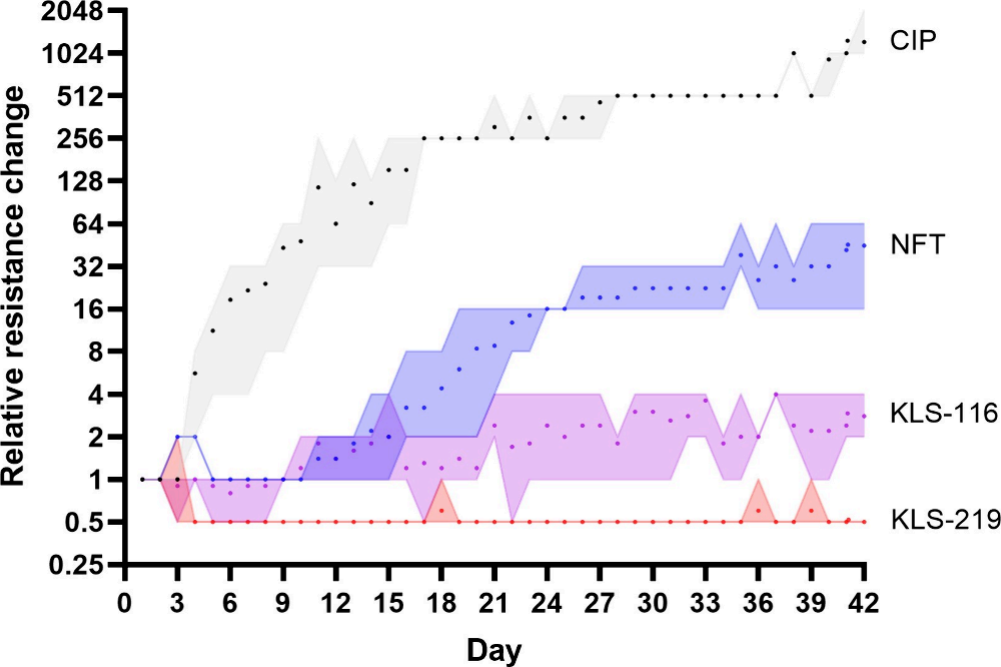
Evolution of resistance to KLS complexes compared to ciprofloxacin and nitrofurantoin *E. coli* was serially passaged in the presence of 0.25× to 8× MIC of each compound for 42 days. Starting MICs were ciprofloxacin: 0.016 mg L^–1^, nitrofurantoin: 4 mg L^–1^, KLS-116: 0.78 μM, and KLS-219: 2.34 μM. Fresh MICs were measured every 24 h, and drug concentrations were adjusted accordingly. Five independent biological replicates were performed per compound. *E. coli* was tested against ciprofloxacin (CIP), nitrofurantoin (NFT), KLS-116, and KLS-219. Dots represent the mean for each MIC with shaded areas representing the minimal and maximum values across the five evolution lineages.

### Optimization of Growth Media and Antimicrobial Activity of KLS-116

To further investigate the effect of KLS-116 against uropathogens under physiological conditions, its activity in conditions that closely modeled the environment within the urinary tract was then investigated.

To ensure that these downstream assays more accurately reflected physiologically relevant conditions, three artificial urine media (AUM) formulations alongside a chemically defined medium^39^ were first evaluated. This step was critical because infection sites such as the urinary tract present unique nutrient profiles and physicochemical properties that can influence antimicrobial performance. The three artificial urine media tested were Brooks and Keevil (BK),^40^ modified AUM,^41^ and enhanced multipurpose AUM (EMP).^42^ The growth of a panel of clinically relevant uropathogens, including members of the ESKAPE group (*Staphylococcus aureus*, *Klebsiella pneumoniae*, *Acinetobacter baumannii*, *Pseudomonas aeruginosa*, and *Enterobacter* spp.), as well as *Escherichia coli*, *Proteus mirabilis*, and *Serratia marcescens*, in these media was first investigated. As these species represent the most common causative agents of catheter-associated urinary tract infections (CAUTIs), establishing an appropriate medium for them not only provides realistic growth conditions but also ensures an accurate assessment of KLS-116 activity for clinical translation.

Our initial screen revealed that the BK medium was not an appropriate model for such studies as it induced substantial precipitation of KLS-116, while modified AUM was not suitable as it only supported limited bacterial growth, particularly for *E. coli*, *P. mirabilis*, and *S. marcescens*. In contrast, EMP supported robust growth across all species and had a minimal effect on the solubility of KLS-116, making it the most physiologically relevant medium for subsequent experiments.

To compare compound performance under physiologically relevant conditions, we next quantified antimicrobial activity in the selected media. Minimum inhibitory and bactericidal concentrations determined in EMP were broadly comparable to those obtained in the chemically defined medium, CDM, with MIC values for both compounds generally ≤25 μM (Table 2). Notably, *S. aureus* exhibited substantial resistance to killing relative to its minimum inhibitory concentration. Based on these findings, EMP was selected as the artificial urine medium for downstream assays.

**Table 2:** Minimum Inhibitory Concentrations (MIC) and Minimum Bactericidal Concentrations (MBC) of KLS-116 against Clinically Relevant Uropathogens in Different Media^a^

	chemically defined media (CDM)		Brooks and Keevil AUM (BK)		modified AUM (mAUM)		enhanced multipurpose urine (EMP)	
bacterial strain	MIC	MBC	MIC	MBC	MIC	MBC	MIC	MBC
A. baumannii NUH: 19Y000374	3.65	3.13	3.13	4.69	1.56	1.82	6.25	6.25
K. pneumoniae NUH: 18Y001710	3.65	2.08	12.50	4.69	3.13	1.82	25.00	75.00
K. quasipneumoniae NUH: 18Y000138	6.25	6.25	12.50	22.92	1.17	1.82	12.50	9.38
S. aureus NUH: 18Y001559	3.13	3.13	6.25	12.50	NG	NG	0.78	0.78
E. hormaechei NUH: 19Y000094	6.25	7.29	12.50	16.67	3.13	11.46	6.25	41.67
P. aeruginosa NUH: 19Y000086	6.25	9.38	12.50	33.33	4.69	3.13	6.25	25.00
E. coli NUH: 17Y000067	6.25	6.25	1.56	18.75	NG	NG	6.25	3.65
P. mirabilis NUH: 18Y000286	12.50	12.50	25.00	25.00	NG	NG	12.50	58.33
S. marcescens NUH: 18Y000153	25.00	25.00	NG	NG	NG	NG	12.50	100.00

a Strains were obtained from the Nottingham University Hospitals (NUH) NHS Trust Pathogen Bank. MIC assays were conducted according to the EUCAST guidelines. NG: no growth, indicating that the medium does not support bacterial growth. Data represent the mean of three biological repeats, with each biological repeat comprising two technical replicates (for standard deviations, see Table S1).

To assess whether the broad-spectrum activity of KLS-116 extended beyond uropathogens, a panel of clinical *Salmonella* isolates, representing enteric pathogens of significant clinical relevance, were also included. Strikingly, all isolates were highly susceptible (MIC 0.6–1.0 μM; Supplementary Table S2), suggesting potential for wider application against Gram-negative infections, including those originating in the gastrointestinal tract.

The selectivity index (SI), defined as CC_50_/MIC,^43^ was calculated using previously reported cytotoxicity data in HEK293 cells (IC_50_ = 135 μM^30^) and antibacterial activity determined under standard Mueller–Hinton broth (MHB) conditions. For *E. coli* EC958, this gives an SI of approximately 84 (MIC = 1.6 μM), indicating a high degree of selectivity for bacterial over mammalian cells.

Expanding on this, we evaluated the antimicrobial activity across a panel of clinically relevant strains in multiple media types following EUCAST-aligned methodologies. As expected, MIC values varied depending on media composition, leading to a corresponding range of SI values across strains and conditions (Table S3). This variability reflects known effects of environmental conditions on antimicrobial activity, while overall SI values remain consistent with favorable selectivity.

### Activity of KLS-116 against Established Biofilms

As discussed above, biofilms present a major barrier to successful treatment of catheter-associated urinary tract infections as they greatly enhance tolerance toward antibiotics, increase bacterial virulence, and consequently have a deleterious effect on successful clinical outcomes. Therefore, given the promising planktonic activity of KLS-116 and its compatibility with conventional antibiotics, its efficacy against mature biofilms was evaluated.

In these studies, the activity of KLS-116 was compared with colistin, a last-resort antibiotic for multidrug-resistant Gram-negative infections, using a modified minimum biofilm eradication concentration (MBEC) tubing assay against 24-h established *Klebsiella quasipneumoniae* biofilms (Figure 4). This species, which is frequently misidentified as *K. pneumoniae* in routine diagnostics, was selected due to its sensitivity to colistin and as a clinically isolated catheter-associated urinary tract infection pathogen that exhibits high levels of multidrug resistance, making it an important challenge organism for biofilm eradication studies.^44^ Biofilms were grown on catheter-like tubing segments in EMP and subsequently exposed to KLS-116 at 1×, 4×, 16×, 64×, or 256× the MIC for 2, 6, or 24 h.

**Figure 4:**
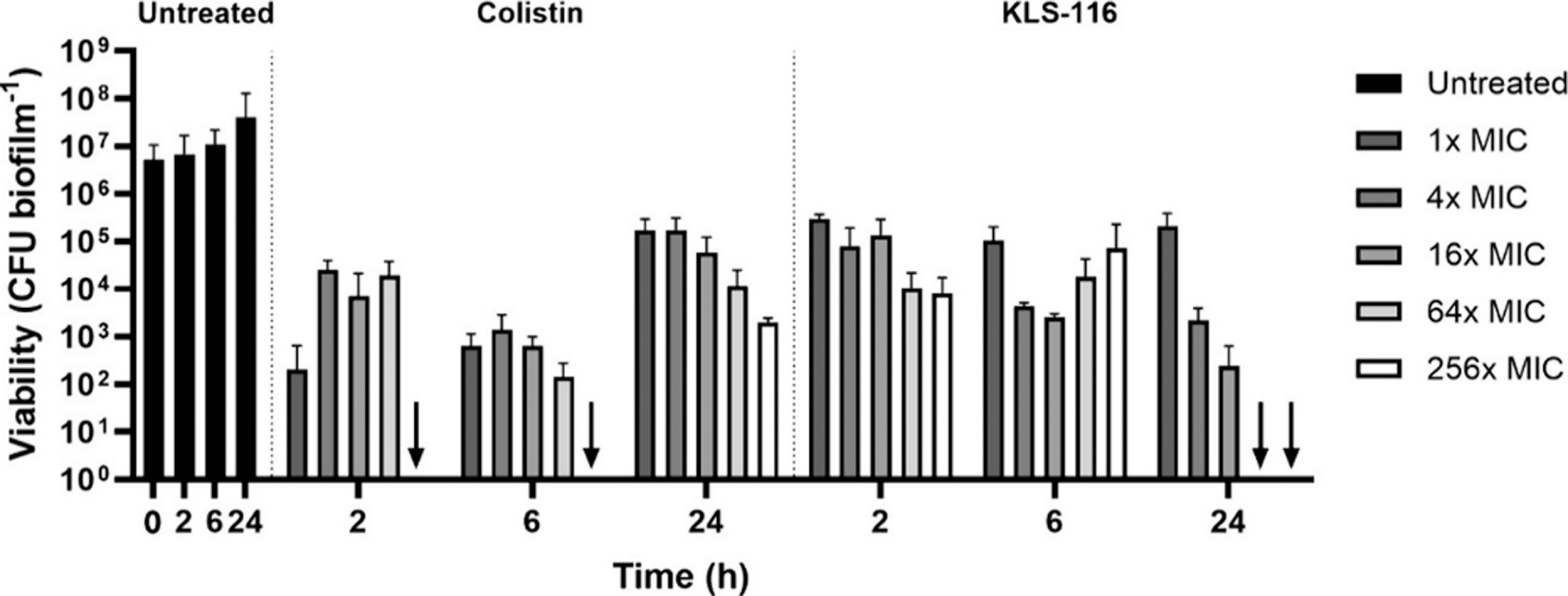
KLS-116 demonstrates substantial reduction of *Klebsiella quasipneumoniae* biofilms in physiologically relevant enhanced multipurpose artificial urine medium using a modified MBEC tubing assay. Biofilms were established for 24 h at 37 °C in artificial urine medium prior to treatment at concentrations ranging from 1× to 256× the MIC of colistin or KLS-116 in fresh medium. Samples were collected at 2, 6, and 24 h post-treatment. The detection limit of the assay was 100 CFU biofilm^–1^. Data represent five biological replicates (mean ± SD).

After 2 h of treatment, all three agents achieved ≥2-log reductions in viable cell counts at the highest concentrations (64× and 256× MIC), with colistin killing biofilm encased cells to below the limit of detection of the assay (100 colony forming units per biofilm) at 256× MIC at 2 and 6 h but with recovery observed at 24 h. At lower concentrations (1–16× MIC), colistin demonstrated greater early activity than KLS-116. However, by 6 h, KLS-116 exhibited increased activity, achieving ≥2-log reduction in viable cells from 4× MIC onward.

Following 24 h of exposure of 1× MIC, both colistin and KLS-116 achieved approximately 2-log reduction in viable cells. At 4× MIC, KLS-116 outperformed colistin, producing a ∼4-log reduction, and at 64× MIC and above, KLS-116 completely eradicated biofilms, reducing counts below the detection limit of the assay.

These findings highlight the potent, concentration-, and time-dependent antibiofilm activity of KLS-116, which exceeded that of colistin after prolonged exposure.

### Evaluation of KLS-116 in a Continuous-Flow Drip-Flow Biofilm Model

To extend these biofilm studies to a more clinically relevant setting, the activity of KLS-116 against mature *K. quasipneumoniae* biofilms using a modified CDC drip-flow reactor was assessed (Figure 5). Unlike the static MBEC assay, this system simulates catheter-associated infections by maintaining a continuous flow of artificial urine medium, promoting the development of dense, resilient biofilms.

**Figure 5:**
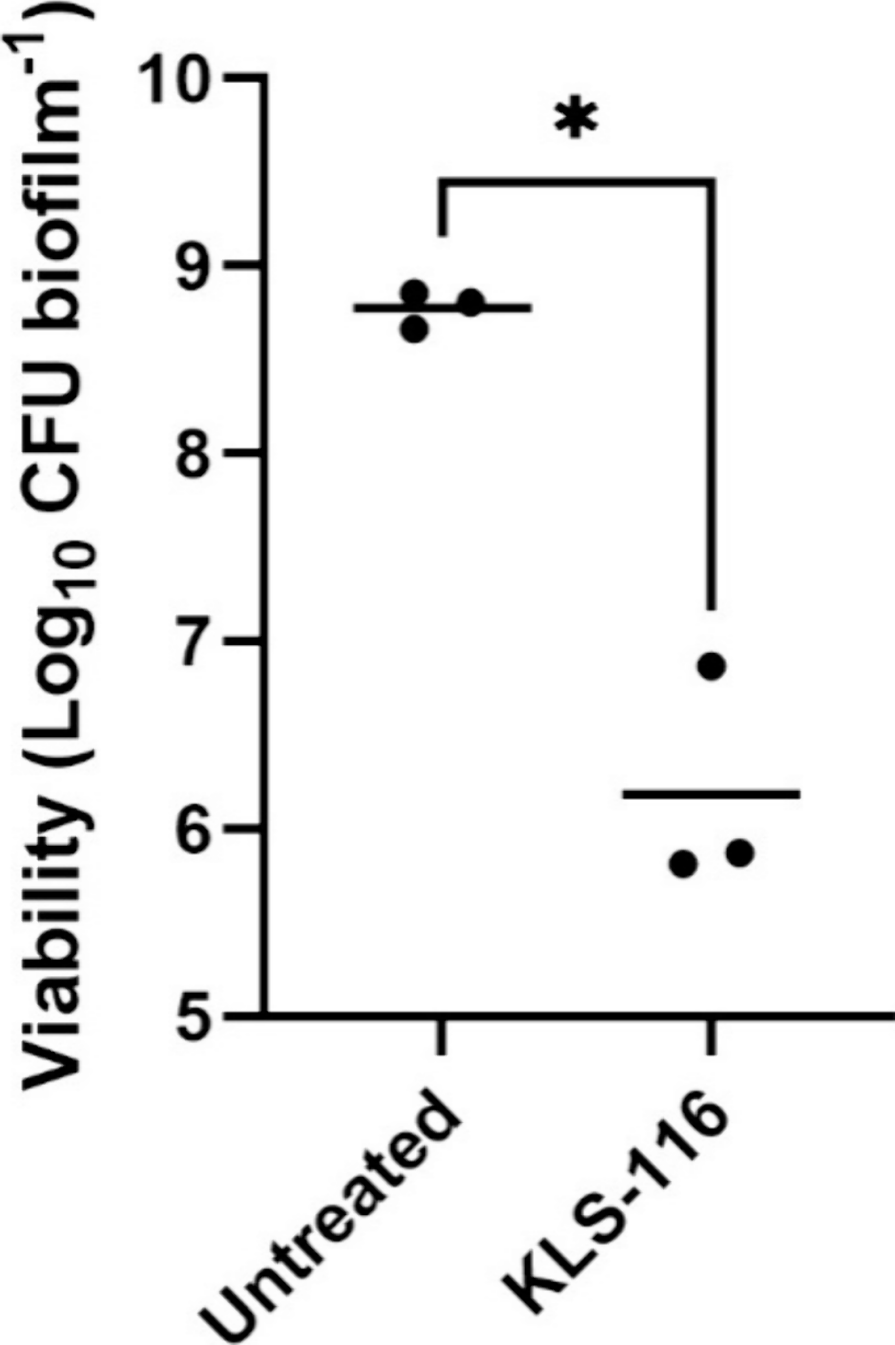
KLS-116 demonstrates a ∼2-log reduction in a five-day established *Klebsiella quasipneumoniae* biofilm grown in artificial urine medium following 24 h of treatment at 4× the MIC. The line represents the mean from three biological repeats. *P = 0.0148, Welch’s *t* test (after the Shapiro–Wilk test for normality).

Biofilms were established over 5 days under continuous flow (0.5 mL min^–1^), after which tubing segments were treated for 24 h with KLS-116 at 4× MIC or with AUM alone (control). KLS-116 achieved a >2-log reduction in viable biofilm-associated bacteria compared with untreated controls (Figure 5). For comparison, 24-h biofilms treated under static conditions in the MBEC assay exhibited a 4-log reduction (Figure 4), highlighting the increased tolerance of mature biofilms formed under flow.

These findings demonstrate that KLS-116 retains significant antibiofilm activity under conditions that closely mimic *in vivo* catheter environments and may also reduce the planktonic bacterial burden within the system.

## Discussion

A major limitation in antimicrobial development is the frequent loss of efficacy when compounds are transitioned from standard laboratory conditions to the complex environments encountered *in vivo*. This includes altered pH, host-specific nutrient composition, and biofilm-associated growth, which is the predominant mode of bacterial persistence in urinary tract infections. By incorporating these parameters at the *in vitro* stage, the present study seeks to identify potential failure modes early and thereby reduce the risk of costly attrition at later stages of the clinical development pipeline. The findings presented in this study highlight several critical aspects of KLS-116 that inform its potential as a next-generation antimicrobial. Beyond demonstrating broad-spectrum activity against multidrug-resistant pathogens, our results provide insight into its mechanism of action and compatibility with existing therapies. Additive interactions and the absence of antagonism with the antibiotics tested, particularly with Meropenem, strengthen its translational potential, as combination therapy, which remains a cornerstone for managing multidrug-resistant infections. These attributes, combined with its stability under physiologically relevant pHs, position KLS-116 as a promising candidate for addressing unmet needs in the treatment of systemic infections.^33–35^

Against this backdrop, the relatively consistent activity of KLS-116 across a broad pH range is notable. The retention of antimicrobial activity between pH 5 and 9 suggests that KLS-116 may be less constrained by the physicochemical and physiological limitations that affect many existing antibiotic classes. This property may be advantageous in infection environments where local pH is variable or deviates from neutrality, such as in biofilm-associated infections of the urinary tract.

While detailed mechanistic experiments were beyond the scope of the present study, the mechanism of action of KLS-116 has been investigated extensively in our previous work. Across multiple studies, KLS-116 has consistently been shown to exhibit antibacterial activity through interactions with the bacterial cell envelope rather than through inhibition of a specific intracellular target.^30–32,39^ Using a combination of electron microscopy, fluorescence-based membrane integrity assays, flow cytometry, and nanoscale biophysical techniques, these studies demonstrated that KLS-116 induces membrane perturbation, loss of barrier function, and rapid bactericidal effects.^30–32,39^ These findings support a membrane-associated mechanism of action for KLS-116, characterized by physical disruption of bacterial membranes and leakage of intracellular components. Such mechanisms are generally less sensitive to environmental variables, including extracellular pH, than target-specific antibiotics. This provides a plausible mechanistic framework for the robust antibacterial activity of KLS-116 observed across a broad pH range in the current study and supports its continued evaluation under physiologically and clinically relevant conditions.

Cytotoxicity and preliminary *in vivo* tolerability of KLS-116 were also evaluated previously.^30^ In particular, human cell viability studies using HEK293 cells demonstrated low cytotoxicity at concentrations exceeding those required for antibacterial activity, indicating a favorable selectivity window. In addition, assessment in the *Galleria mellonella* infection model showed that KLS-116 was well tolerated *in vivo* at therapeutically relevant doses while retaining antibacterial efficacy.^30^ While further comprehensive mammalian *in vivo* toxicity and pharmacokinetic studies remain necessary, these data provide an important early indication of host compatibility and support continued preclinical evaluation of KLS-116.

In addition to demonstrating host tolerability, KLS-116 also exhibited clear antibacterial efficacy *in vivo* in the *Galleria mellonella* infection model.^30^ Treatment with KLS-116 significantly improved survival of larvae infected with clinically relevant bacterial pathogens, indicating effective bacterial clearance at therapeutically relevant doses. This provides early evidence that KLS-116 retains antibacterial activity in a living host environment and supports its further evaluation in more advanced mammalian infection models.

Building on these previous findings, including antibacterial efficacy studies, the long-term evolution assay described in this report was used to assess the propensity for resistance development to KLS-116 under prolonged selective pressure. Strikingly, over 42 days of continuous passaging under increasing selective pressure, neither KLS-116 nor its mononuclear analogue KLS-219 developed any meaningful or sustained increases in resistance. The control antibiotics displayed expected trends, with ciprofloxacin rising rapidly in resistance and nitrofurantoin showing a slower, more constrained increase,^45–47^ confirming that the system was capable of detecting evolutionary responses when they occurred. Notably, the slower evolutionary trajectory of nitrofurantoin is widely attributed to its multimodal mechanism of action, which aligns with the limited evolutionary adaptability seen for the KLS complexes.^47^ The inability of *E. coli* to acquire stable resistance across this extended period of time suggests that the KLS complexes may target multiple cellular pathways and implies that resistance emergence in clinical use may be slower than that of traditional antibiotics.

Another major barrier to effective clinical translation of antimicrobial agents is the failure to eradicate biofilm-associated infections, which are inherently more robust and tolerant to treatment than planktonic populations. This challenge is particularly acute in catheter-associated urinary tract infections, where biofilm formation on indwelling devices is the primary driver of persistence, recurrence, and therapeutic failure.^48^ In this context, the ability of KLS-116 to eradicate mature biofilms in both static and continuous-flow models suggests effective penetration of the biofilm matrix and sustained activity against sessile cells, which typically exhibit reduced metabolic rates and increased tolerance to conventional antibiotics.^49^ Eradication of established *K. quasipneumoniae* biofilms under both static and continuous-flow conditions represents a significant advance over many conventional antibiotics, which often fail in these settings.^50^ In the modified MBEC assay, KLS-116 achieved complete clearance of 24-h biofilms at concentrations ≥16× MIC, outperforming colistin after prolonged exposure. Importantly, these findings were corroborated in a drip-flow reactor model that mimics the dynamic environment of indwelling urinary catheters. Although the reduction in viable cells was lower than in static assays (>2-log vs ∼4-log), this is consistent with the increased resilience of mature biofilms formed under continuous nutrient flow.

These findings position KLS-116 as a robust antimicrobial candidate whose retained activity under clinically relevant conditions supports progression toward advanced *in vivo* evaluation for (catheter-associated) UTI infections. Its broad-spectrum activity against multidrug-resistant uropathogens, stability across physiologically relevant pH ranges, and indifference/additive interactions with standard antibiotics support potential use in systemic therapy and device-associated infection management. Importantly, the ability to eradicate mature biofilms in both static and continuous-flow models addresses a critical unmet need in catheter-associated urinary tract infections.^12^ Future work will focus on optimizing delivery strategies such as catheter coatings or localized release systems, validating efficacy and safety in *in vivo* models to bridge the gap toward clinical application, and conducting transcriptomic analyses to determine how bacteria adapt at the molecular level to KLS-116 exposure. Such studies will be important for identifying stress responses, potential resistance pathways, and opportunities for rational combination therapy, thereby supporting long-term efficacy and minimizing resistance development. While comprehensive mammalian biocompatibility and pharmacokinetic studies remain essential future steps, the combined tolerability data reported previously and the robust antibacterial performance demonstrated here support the continued preclinical development of KLS-116. We note that plasma stability and protein-binding effects were not directly assessed in this study; however, the retention of activity in complex media over extended periods (up to several months) is consistent with functional stability, and dedicated pharmacokinetic evaluation will be required in future work.

## Materials and Methods

### Bacterial Strains and Media Used

Growth was achieved in Mueller–Hinton broth, glucose defined minimum medium,^30^ chemically defined minimal medium,^39^ or one of three types of artificial urine media,^40–42^ made up as per manufacturer’s instructions or as described within the literature. Enhanced multipurpose artificial urine medium was made with 0.1% peptone and 0.0005% yeast extract.^42^ Overnight cultures were prepared by inoculating a single colony into 5 mL of appropriate sterile medium and incubation at 37 °C with shaking at 200 rpm for 18 h. Bacterial strains were obtained from Nottingham University Hospitals Trust Pathogen Bank, UK (Table 1).

### Preparation and Storage of KLS-116 and Antibiotics

The complex was synthesized as previously described.^30^ Stock solutions were made to a concentration of 5 mg mL^–1^ in sterile deionized water and stored at room temperature protected from light.

Ciprofloxacin was solubilized in 0.1 M hydrochloric acid (HCl, Fisher, USA) to a concentration of 20 g L^–1^ and stored at −20 °C. Nitrofurantoin was made fresh daily by solubilization in dimethyl sulfoxide (DMSO, Fisher, USA) to a concentration of 10 g L^–1^. Both antibiotics were sterile filtered through 0.22 μM polyether sulfone filters (Starlab, USA).

### Minimal Inhibitory and Bactericidal Concentration Assays

Minimum inhibition concentration (MIC) assays were performed as previously described.^32^ To determine the minimum bactericidal concentration (MBC), 10 μL from each well showing no visible growth was subcultured onto Mueller–Hinton agar plates alongside a positive growth control. Plates were incubated at 37 °C for 18 h, and the lowest concentration that yielded no colony growth was recorded as the MBC.

### Checkerboard Microdilution Assays

Checkerboard assays were performed as previously described to assess interactions between KLS-116 and conventional antibiotics. Fractional inhibitory concentration indices (FICI) were interpreted as follows: synergy (≤0.5), additive (>0.5–1.0), no interaction/indifference (>1.0–4.0), and antagonism (>4.0).^51^

### Modified Minimum Biofilm Eradication Concentration (MBEC) Assay

Thermoplastic polyurethane (Saint Gobain Versilon C-210-A, internal diameter 6.4 mm, outer diameter 9.6 mm, length 1.5 cm) tubing was affixed to the underside of 24-well plate lids using cyanoacrylate adhesive (Everbuild Stick2). Plates were air-dried overnight and UV-sterilized for 20 min prior to use. Overnight bacterial cultures were diluted in fresh medium to an OD_600_ of 0.1. Each well was filled with 1.8 mL of medium and inoculated with 200 μL of the diluted suspension. Lids with affixed tubing segments were replaced so that the tubing was fully submerged without contacting the well bottom. Plates were incubated at 37 °C on a bed shaker (70 rpm) for 24 h to allow biofilm formation.

Biofilm eradication was assessed by exposing tubing segments to antibiotic concentrations ranging from 1× to 256× the MIC, prepared in sterile medium. Tubes were challenged for 2, 4, or 24 h. Pretreatment tubing controls and wells containing fresh medium for equivalent incubation periods were included.

Following treatment, tubing segments were removed, rinsed gently with 1 mL phosphate-buffered saline by shaking for 10 s, and transferred to fresh 1 mL of PBS for biofilm removal. Biofilms were detached as previously described.^52^ Briefly, the following processes were performed: vortexing for 30 s at 2000 rpm, sonication at 40 kHz and 37 °C for 5 min, followed by an additional 30 s of vortexing. Suspensions were serially diluted in PBS and plated on Mueller–Hinton agar for viable count enumeration.

### Modified Drip Flow Biofilm Reactor Assay

A modified four-channel Drip Flow Biofilm Reactor (DFR, Biosurface Technologies Corp., USA) was used to assess the activity of KLS-116 against mature *K. quasipneumoniae* biofilms under continuous-flow conditions. Size 16 tubing was used throughout the system, with 7.6 cm segments fitted into each channel. Channels were operated in biologically matched pairs: channels 1 and 2 were inoculated with one overnight culture and channels 3 and 4 with a second. Within each pair, one channel received KLS-116 treatment, and the other served as an untreated control.

Peristaltic pump settings were calibrated to deliver a flow rate of 0.5 mL min^–1^ per channel, with a maximum error of ±0.068 mL min^–1^ across the system. A 500 mL exponential-phase bacterial suspension was prepared and flowed through the reactor at 0.5 mL min^–1^ per channel for 2 h at room temperature to initiate colonization. Sterile, prefilled 5 L bottles containing EMP medium were connected to the system using glass breaks to prevent backflow. The reactor was transferred to a warm room, and biofilms were allowed to mature over a 5-day period under continuous flow (0.5 mL min^–1^ per channel).

Fresh EMP medium reservoirs were prepared in the presence or absence of KLS-116. Treatment was applied at 4× the MIC for *K. quasipneumoniae* (50 μM). Channels were treated for 24 h at a flow rate of 0.5 mL min^–1^. Following treatment, tubing segments were aseptically removed and washed by pipetting 2 mL of PBS through the lumen. Tubes were transferred to 20 mL of PBS, and the biofilm was removed, and viable cells were quantified as described for MBEC assays.

### Antimicrobial Evolution Assay

Initial cultures were pregrown in GDMM to generate a uniform starting inoculum for all experimental conditions. For each antimicrobial compound, evolutionary selection was performed in 24-well microtiter plates (Sarstedt) using a six-well, 2-fold dilution gradient per evolutionary line. Drug concentrations were arranged such that the previously determined minimum inhibitory concentration (MIC) was positioned in well 4 of the six-well series. Wells 1–3 contained 2-fold increases above the MIC (up to 8× MIC), and wells 5 and 6 contained 0.5× and 0.25× MIC, respectively. Drug-free controls were passaged in parallel under identical conditions to monitor spontaneous resistance emergence in the absence of selection.

Each well was inoculated with 1% (v/v) of an overnight culture and incubated statically for 24 h. Following incubation, the well showing visible growth at the highest antimicrobial concentration was identified. This culture was used (i) to estimate the updated MIC for that evolutionary line for the subsequent passage and (ii) as the inoculum source for the next passage. The antimicrobial gradient was then recalculated for the following day based on the updated MIC, maintaining the same six-well layout (8×, 4×, 2×, 1×, 0.5×, 0.25× MIC). This procedure was repeated daily, enabling longitudinal tracking of resistance across sequential passages.

Five independent evolutionary lines were propagated for each compound, alongside six independent drug-free control lines. The evolution experiment was performed for 42 daily passages. To preserve evolutionary trajectories, cultures from each line were archived daily in glycerol stocks and stored at −80 °C.

## Supplementary Material



## Notes

Table S1. Standard deviations for MIC and MBC for KLS-116 against clinically relevant uropathogens in different media. Table S2. Minimum inhibitory concentrations (MICs) of KLS-116 against clinical Salmonella isolates. Table S3. Selectivity index values of KLS-116 against clinically relevant uropathogens in different media (PDF)

## †

The Department of Clinical Sciences, Liverpool School of Tropical Medicine, Pembroke Place, Liverpool L3 5QA, UK (B.J.)
