# Supplementary material for: Broad-Spectrum Antimicrobial Activity of a Ruthenium(II) Polypyridyl Complex against Multidrug-Resistant Uropathogens and Biofilms
Source: ACS Omega. 2026 Jun 18;11(25):37874–83. doi: 10.1021/acsomega.6c03019 (PMC13325168; doi:10.1021/acsomega.6c03019)
Supplement: Supplementary file 1 [file ao6c03019_si_001.pdf]

Broad-spectrum antimicrobial activity of a ruthenium(II) polypyridyl complex against multidrug-resistant uropathogens and biofilms

Beth James[1], Simon D Fairbanks[2], Mia Horton[3], Jim A Thomas[2*], Samantha McLean[3] and Adam M Varney[1*]

[1]Medical Technologies Innovation Facility (MTIF), Nottingham Trent University, Clifton Lane, Nottingham, NG11 8NS UK.

[2]Department of Chemistry, University of Sheffield, Brook Hill, Sheffield, S3 7HF, UK

[3]School of Science and Technology, Nottingham Trent University, Clifton Lane, Nottingham, NG11 8NS, UK

*Corresponding authors

Dr Adam Varney (microbiology) E-mail: adam.varney@ntu.ac.uk

Professor James A Thomas (chemistry) E-mail: james.thomas@sheffield.ac.uk

Supplementary information

Table S1. Standard deviations for MIC and MBC for KLS-116 against clinically relevant uropathogens in different media.

Bacterial strain	Chemically Defined Media (CDM)		Brooks & Keevil AUM (BK)		Modified AUM (mAUM)		Enhanced Multipurpose Urine (EMP)	
	MIC	MBC	MIC	MBC	MIC	MBC	MIC	MBC
A. baumannii NUH: 19Y000374	1.80	0.00	1.18	1.71	0.00	0.64	0.00	0.00
K. pneumoniae NUH: 18Y001710	1.80	0.81	4.72	1.71	0.00	0.64	0.00	27.39
K. quasipneumoniae NUH: 18Y000138	2.36	0.00	4.72	5.10	0.43	0.64	0.00	3.42
S. aureus NUH: 18Y001559	1.18	0.00	2.36	0.00	NG	NG	0.00	NG
E. hormaechei NUH: 19Y000094	2.36	2.55	4.72	6.45	0.00	2.55	0.00	12.91
P. aeruginosa NUH: 19Y000086	2.36	3.42	4.72	12.91	1.71	0.00	0.00	0.00
E. coli NUH: 17Y000067	2.36	0.00	0.59	6.85	NG	NG	0.00	1.28
P. mirabillis NUH: 18Y000286	4.72	0.00	9.45	NG	NG	NG	0.00	20.41
S. marcescens NUH: 18Y000153	9.45	0.00	NG	NG	NG	NG	0.00	0.00

Strains were obtained from the Nottingham University Hospitals (NUH) NHS Trust Pathogen Bank. MIC assays were conducted according to EUCAST guidelines. NG indicates no growth, meaning the medium was unable to support bacterial growth. Data represent the standard deviation of three biological repeats, with each biological repeat comprising two technical replicates

Table S2. Minimum inhibitory concentrations of KLS-116 against clinical *Salmonella* isolates.

MIC values (µM) were determined in gDMM following 18 h incubation at 37 °C. Data represent the mean ± SD of three biological replicates. The selectivity index (SI), defined as CC_{50}/MIC, was calculated using previously reported cytotoxicity data in HEK293 cells (IC_{50} = 135 µM; Smitten et al., ACS Nano 2019)

Bacterial strain	MIC (SD) µM	Selectivity Index (SI)
Salmonella sp. NUH: 19Y000277	0.6 (0.2)	225
Salmonella sp. NUH: 19Y000355	0.7 (0.2)	192.9
Salmonella sp. NUH: 19Y000431	0.8 (0.3)	168.8
Salmonella sp. NUH: 19Y000453	0.7 (0.2)	192.9
Salmonella sp. NUH: 19Y000469	0.8 (0.3)	168.8
Salmonella sp. NUH: 20Y000068	0.7 (0.4)	192.9
Salmonella sp. NUH: 20Y000082	0.8 (0.3)	168.8
Salmonella sp. NUH: 20Y000088	0.8 (0.3)	168.8
Salmonella sp. NUH: 20Y000124	1.0 (0.4)	135
Salmonella sp. NUH: 20Y000195	0.8 (0.3)	168.8

Table S3. Selectivity Index values of KLS-116 against clinically relevant uropathogens in different media.

The selectivity index (SI), defined as CC_{50}/MIC, was calculated using previously reported cytotoxicity data in HEK293 cells (IC_{50} = 135 μM; Smitten et al., ACS Nano 2019)

		Selectivity Index (SI)			
		Chemically Defined Media (CDM)	Brooks & Keevil AUM (BK)	Modified AUM (mAUM)	Enhanced Multipurpose Urine (EMP)
A. baumannii	NUH: 19Y000374	36.99	43.13	86.54	21.60
K. pneumoniae	NUH: 18Y001710	36.99	10.80	43.13	5.40
K. quasipneumoniae	NUH: 18Y000138	21.60	10.80	115.38	10.80
S. aureus	NUH: 18Y001559	43.13	21.60	NA	173.08
E. hormaechei	NUH: 19Y000094	21.60	10.80	43.13	21.60
P. aeruginosa	NUH: 19Y000086	21.60	10.80	28.78	21.60
E. coli	NUH: 17Y000067	21.60	86.54	NA	21.60
P. mirabillis	NUH: 18Y000286	10.80	5.40	NA	10.80
S. marcescens	NUH: 18Y000153	5.40	NA	NA	10.80
